# CPEB and miR-15/16 Co-Regulate Translation of Cyclin E1 mRNA during *Xenopus* Oocyte Maturation

**DOI:** 10.1371/journal.pone.0146792

**Published:** 2016-02-01

**Authors:** Ania Wilczynska, Anna Git, Joanna Argasinska, Eulàlia Belloc, Nancy Standart

**Affiliations:** 1 Department of Biochemistry, University of Cambridge, Cambridge, United Kingdom; 2 Cancer Research UK Cambridge Institute, University of Cambridge, Cambridge, United Kingdom; 3 Institute for Research in Biomedicine, Barcelona, Spain; University of Surrey, UNITED KINGDOM

## Abstract

Cell cycle transitions spanning meiotic maturation of the *Xenopus* oocyte and early embryogenesis are tightly regulated at the level of stored inactive maternal mRNA. We investigated here the translational control of cyclin E1, required for metaphase II arrest of the unfertilised egg and the initiation of S phase in the early embryo. We show that the cyclin E1 mRNA is regulated by both cytoplasmic polyadenylation elements (CPEs) and two miR-15/16 target sites within its 3’UTR. Moreover, we provide evidence that maternal miR-15/16 microRNAs co-immunoprecipitate with CPE-binding protein (CPEB), and that CPEB interacts with the RISC component Ago2. Experiments using competitor RNA and mutated cyclin E1 3’UTRs suggest cooperation of the regulatory elements to sustain repression of the cyclin E1 mRNA during early stages of maturation when CPEB becomes limiting and cytoplasmic polyadenylation of repressed mRNAs begins. Importantly, injection of anti-miR-15/16 LNA results in the early polyadenylation of endogenous cyclin E1 mRNA during meiotic maturation, and an acceleration of GVBD, altogether strongly suggesting that the proximal CPEB and miRNP complexes act to mutually stabilise each other. We conclude that miR-15/16 and CPEB co-regulate cyclin E1 mRNA. This is the first demonstration of the co-operation of these two pathways.

## Introduction

Regulation of gene expression at the level of translation is pivotal in early development, particularly during meiotic maturation of the oocyte and early embryogenesis, when transcription is shut down. During these periods, stored maternal mRNA, held in a translationally quiescent state in the oocyte, is mobilised into polysomes to provide the egg and embryo with critical cell cycle regulatory factors. The targets, as well as the cis- and trans-acting players involved in such control mechanisms, have been especially well-studied in *Xenopus laevis*. *Xenopus* (stage VI) oocytes, arrested in prophase of meiosis I, can be triggered to undergo meiotic maturation (GVBD or germinal vesicle breakdown) with progesterone, and laid eggs can be readily fertilised to generate embryos, which only initiate zygotic transcription after the 12^th^ cell division (mid-blastula transition).

Work in several laboratories has shown that a major regulator of translation in *Xenopus* oocytes and eggs is CPEB, which mediates both translational repression in the oocytes and subsequently translational activation in the egg [1, 2]. CPEB, an RRM and Zinc finger-containing protein, interacts with short U-rich elements, called cytoplasmic polyadenylation elements (CPEs), typically U_4_AU, in the 3‘ UTR of regulated mRNAs such as those encoding cyclin B1 and c-mos. Binding of CPEB to CPEs in target mRNAs in oocytes results in the formation of a repressed closed-loop of mRNA, with the cap-binding eIF4E protein prevented from productive interaction with eIF4G to recruit the small ribosomal subunit to initiate translation. eIF4E-binding factors, which interact directly or indirectly with CPEB to form this loop have been characterised, including 4E-T (reviewed [3]). Meiotic maturation results in CPEB phosphorylation leading eventually to its proteolysis shortly after GVBD. Prior to GVBD, CPEB phosphorylation is proposed to both allow release of co-repressors and the stable association with the GLD-2 poly(A) polymerase to promote cytoplasmic polyadenylation of the repressed mRNAs, resulting in efficient translation [4–11]. Additional 3’UTR-binding protein factors, including Musashi and Pumilio, co-regulate mRNA poly(A) tail lengths and translation with CPEB in meiosis [11, 12]. Regulation of gene expression by polyadenylation in *Xenopus* eggs is enabled by the absence of decapping activity in early development till the mid-blastula transition, ensuring the stability of maternal mRNAs with short poly(A) tails for months in the ovary [13, 14]. In contrast, in somatic cells, such mRNAs would rapidly undergo decapping, and then decay [15].

miRNAs, short ~22 nt non-coding RNAs that are processed from longer hairpin-containing transcripts by Drosha and Dicer nucleases, bind to mRNA typically in their 3’UTR, and also regulate translation and/or mRNA decay. Complementarity to the 5′ end of the miRNA—the 'seed' sequence, containing nucleotides 2–7—is a major determinant in target recognition and is sufficient to trigger silencing. microRNA silencing of target mRNAs is mediated by the multiprotein complex miRNP/miRISC, which includes the key protein components Argonaute 2 and GW182 (TNRC6A-C in humans) (reviewed [16, 17]). According to several recent studies, microRNAs first mediate translational repression, at the level of initiation, and subsequently lead to deadenylation and decay of their target mRNAs [18–21].

Previously, we and others have identified microRNAs in *Xenopus laevis* and *tropicalis* oocytes by high-throughput sequencing, and verified their presence by Northern blot analysis [22–24]. MicroRNAs have also been identified in mouse oocytes, and initial studies showed that mouse Dicer -/- oocytes fail to make the meiosis I-II transition and to progress through the first cell division, suggesting an important early developmental role for maternal microRNAs [25, 26]. In contrast, more recent reports suggest that miRNA function is suppressed in murine oocytes [27, 28]. Of note, however, mouse oocytes undergo meiosis I (~10–17 days) in considerably less time than in *Xenopus* (~ 3–5 months), and mouse maternal mRNAs are degraded and replaced by zygotic transcripts at the 2 cell stage, rather that at the12^th^ cell division as in *Xenopus* embryogenesis, (reviewed [29, 30], implying that regulation of maternal mRNA translation may not be as important in early mouse development as it is in *Xenopus*.

Here we focus on miR-15/16, abundant/moderately abundant microRNAs in *Xenopus* oocytes and eggs [22–24]. miR-15 and miR-16 are present in gene clusters and share the same 8 nt seed sequence. The highly-conserved mir-15/16 cluster is frequently deleted in cancer, and in mammalian cell lines silencing by miR-15/16 induces cell cycle arrest in G1/G0. Overexpression of these miRNAs inhibits cell proliferation, promotes apoptosis of cancer cells, and suppresses tumorigenicity both *in vitro* and *in vivo* (reviewed [31, 32]). In other words, miR-15/16 can act as tumor suppressors.

One of the major targets of miR-15/16 in mammalian cells is cyclin E1 mRNA, with two conserved 3’UTR binding sites [33–36]. Interestingly, work in *Xenopus* oocytes, eggs and embryos suggested that cyclin E1 mRNA may be translationally regulated during meiosis [37–39]. Cyclin E1/Cdk2 is required for metaphase II arrest of the unfertilised egg as well as initiation of the S phase in the early embryo [40, 41].

We investigated maternal cyclin E1 mRNA expression, which we demonstrate to be regulated at the level of translation by repression in the oocyte, and cytoplasmic polyadenylation in the egg, mediated both by CPE elements and by miR-15/16 binding sites in its 3’UTR. In line with these reporter studies, mature forms of maternal miR-15/16 were shown to co-immunoprecipitate specifically with the CPEB complex. Sequestering of CPEB protein by pre-injected short CPE-containing RNA reduces miR-mediated repression of reporter mRNA in the oocyte. Strikingly, injection of anti-miR-15/16 LNA results in the early polyadenylation of endogenous cyclin E1 mRNA during meiotic maturation, and an acceleration of GVBD, altogether suggesting the co-occupancy of the CPEB and miRNP/RISC complexes on the mRNA reinforces their activity. Thus, we provide the first evidence of cooperative activity of these two important post-transcriptional pathways.

## Materials and Methods

All experiments involving *Xenopus laevis* were performed in accordance with the Animals (Scientific Procedures) Act 1986 under Schedule 1. Specific details: University of Cambridge committee approval—by AWERB (Animal Welfare & Ethical Review Body) All animals purpose bred for research from commercial sources. Animals are sacrificed using a Home Office approved Schedule 1 method which is a non licensed method. Method, Overdose of anaesthetic by immersion in MS222 for 30 min, followed by destruction of the brain to confirm death. The University Animal Welfare Policy (available on request) details that Animals are transported, housed and cared for by dedicated and trained staff under professional supervision in a manner designed to ensure the best health and well-being of the animal, with provisions for environmental enrichment. Named Veterinary Surgeons are available at all times for consultation, care and attendance. The University of Cambridge is committed to the responsible use of animals in its research and teaching activities. All University personnel who supervise or undertake activities involving animals are trained to carry out their duties in a responsible and humane manner.

### Plasmid constructs and *in vitro* transcription

The plasmids encoding the tethering constructs HA-GW182-ED, NHA-GW-182-ED, Rluc-BoxB used were a kind gift of W. Filipowicz [42]. For *in vitro* transcriptions, plasmids encoding the HA-tagged proteins or the Rluc-BoxB were linearised with *Not* I or *BamH* I, respectively, prior to transcription with T7 RNA polymerase. The *Xenopus tropicalis* Ago2 ORF (NM_001004877) was cloned into pCS2 vector containing an HA tag at the C-terminal end. *Hpa* I linearisation of the pCS2-HAxtAgo2 plasmid was followed by SP6 *in vitro* transcription (Ambion mMessage mMachine kit) and addition of poly(A) tail (Ambion Poly(A) Tailing kit) according to the manufacturer’s instructions. The pIRESneo-FLAG/HA_Ago2_corrected plasmid (Addgene) encoding human Ago2 was linearised with *BamH* I, followed by T7 transcription (Ambion mMessage mMachine kit) and addition of poly(A) tail (Ambion Poly(A) Tailing kit) according to the manufacturer’s instructions.

The cyclin E1 3’UTR was amplified by PCR using primers JA17/18 from the pBluescript plasmid template (a gift from Anne Couturier; [38]) and cloned into firefly Luc-MCS [43] digested with *Sac* I and *Kpn* I, producing Luc-MCS cycE1 WT. To obtain a construct containing mutations in all CPE sequences within the cyclin E1 3’UTR (CPE mut), the LUC-MCS cycE1 plasmid was subjected to sequential QuikChange site-directed mutagenesis (Stratagene) with the following primer pairs: JA21/22, JA23/24, JA 25/26, JA29/30 (For oligonucleotide primer sequences see S1 Table). Site-directed mutagenesis using primer pairs miR-16-1 mut for/miR-16-1 mut rev and miR-16-2 mut for/miR-16-2 mut rev was used to introduce mutations into the seed region of the 1^st^ and 2^nd^ miR-15/16 target sites, respectively (TGCTGCTA>AGCAGGTA). For the construct containing mutations in both miR target sites, the primer pairs were used sequentially. Using LUC-MCS cycE1 WT and LUC-MCS cycE1 CPE mut in these reactions produced miR mut and CPE+miR mut constructs. A *Nhe* I restriction site was introduced immediately upstream of the BamH I site of LUC-MCS using site-directed mutagenesis with primer pair Fluc Ccne1 Nhe mutF/ Fluc Ccne1 Nhe mutR.

For *in vitro* transcription, the plasmid was linearised with Nhe I (to obtain a poly(A)^-^ mRNA) and transcribed using T7 RNA Polymerase. For transcripts containing an (A)_48_ tail, a silent mutation was introduced into the Fluc ORF to remove the *EcoR* I restriction site using site-directed mutagenesis (primer pair Fluc EcoRI mutF / Fluc EcoRI mutR). These plasmids were linearised with *EcoR* I and transcribed using T7 RNA polymerase to obtain poly(A)_50_ transcripts. The luc-400 plasmid (a gift from Dr Lucy Colegrove-Otero) was constructed to contain the antisense sequence of the 290–712 fragment of cloning vector pSP6-T3.

For tethering assays, firefly luciferase mRNA transcribed from the lucMS2 plasmid was used as an internal control. It was linearised with SpeI prior to transcription with T7 RNA polymerase. For firefly luciferase reporter assays, the hRluc mRNA transcribed using T7 RNA polymerase from the phRL-TK linearised with *Not* I was used as a control. *In vitro* poly(A) tailing reactions were carried out as above.

For injection of competitor CPE-containing RNAs, the cyclin B1 3’UTR 65 bp fragment (gauccuaaauaguguauuguguuuuuaauguuuuacugguuuuaauaaagcucauuuuaacaugg) was created by annealing complementary primers JA14/15, which were then cloned into the *Eco*R I/*Bam*H I sites of the pGEM2 vector (Promega). The plasmid was linearized with *Eco*R I (to obtain a poly(A)^-^ transcript) and transcribed using T7 RNA Polymerase. The 3’UTR containing no CPE sequences was derived from the 86 bp wee1 eCPE construct (uuuauugacuuuguuguuuuugguaucuuauugucugguaaauaaaaauuggaaugugua), a gift from A. Charlesworth [44]. Introduction of the eCPE mutation to obtain a sequence without CPEs was performed by mutagenesis using primers JA31/32 (see S1 Table).

### *Xenopus* oocyte and egg lysate preparation

Isolation, staging, handling, and lysate preparation of *Xenopus* oocytes and eggs was performed as previously described [5]. Defoliculated stage VI oocytes were meiotically matured to eggs with the addition of 10 μg/ml progesterone for 20 h or as indicated.

### Luciferase assays

Stage VI oocytes were injected with 0.2 fmol of each of *in vitro* transcribed FLuc reporters and RLuc control mRNAs, and, unless indicated otherwise, the oocytes were incubated for 6 h. 5 pools of 5 oocytes were collected and lysed using 200 μl 1 x PLB (Passive Lysis Buffer, Promega). Luciferase activities were determined with the Dual Luciferase system (Promega) using a Glomax luminometer, as described previously [45]. For tether function assays, 40 fmol of mRNA encoding the λN fusion protein were injected. Fluc control were coinjected. Oocytes were incubated for 6h, lysed and assayed for luciferase expression.

### miRNA inhibitors

LNA inhibitors targeting xtr-miR-16a and -15b (miRCURY LNA Knockdown 173011–00, 173013–00) as well as the miRNA Inhibitor Negative Control A were obtained from Exiqon. For injections, 70 fmol of the miR-16a LNA and 700 fmol of the miR-15b and control LNAs were injected into stage VI oocytes. Optimal concentrations of LNAs were empirically defined by RT-qPCR, as those resulting in near-zero free targeted miRNA and unaffected levels of several unrelated or even partly-homologous miRNAs (data not shown).

### Western Blotting

Western blotting was performed as described before [5] on 10% and 15% SDS-PAGE gels. Primary antibodies used: CPEB (1:2000; [46]), cyclin E1 (1:1000; gift of Tim Hunt) and HA (1:1000; Roche).

### Immunoprecipitation

Fifty nl of 50 ng/μl of the xtAgo2-HA polyA+ RNA were injected into stage VI *Xenopus laevis* oocytes.25 oocytes were used per condition (-progesterone/+ progesterone). Anti-HA-biotin antibodies (Roche) were bound to Dynabeads M-280 streptavidin (Invitrogen) and incubated with oocyte lysates prepared in 150 mM NaCl, 25 mM Tris-HCl pH 7.4, 5 mM EDTA, 0.5% NP40, 5 mM DTT, 1 mM PMSF, 50 μM leupeptin, 50 μM pepstatin, 0.5 μM aprotinin for 2h at 4°C. After washing, bound proteins were eluted with SDS sample buffer, and analysed by western blotting. For immunoprecipitation in the presence of RNAse A, stage VI *Xenopus laevis* oocytes were injected with 46 nl of 500 ng/μl hAgo2-FLAG polyA+ RNA. After 24h 80 oocytes per condition were lysed in NET buffer containing 20 pg/μl RNAse A. Lysate was incubated with 80 μl of FLAG-M2 magnetic beads (Sigma) for 2h 30min at 4°C. After washing, bound proteins were eluted with SDS sample buffer, and analysed by western blotting.

For immunoprecipitation of CPEB followed by RNA extraction, 5 μl CPEB antibody or an isotype matched His-tag antibody (Abcam) was pre-bound to 25 μl protein A Sepharose beads (GE Healthcare) equilibrated in 0.5 ml NET buffer supplemented with 1 mM DTT, 80 μ/ml RNAse inhibitor for 4.5 hours at 4°C with agitation. Beads were washed twice with NET buffer. Lysate of 500 oocytes was pre-cleared using 25 μl protein A Sepharose beads at 4°C for 1 h with agitation in NET buffer. Pre-cleared lysate was added to antibody-bound beads and incubated at 4°C for 2.5 h with agitation in 1 ml NET buffer. Beads were washed 3 times in NET buffer. RNA was extracted from bound beads resuspended in 250 μl of TNES (0.1 M Tris, pH 7.5, 0.3 M NaCl, 5 mM EDTA, 2% SDS) with the addition of 200 μg/ml proteinase K at 50°C for 30 min with intermittent vortexing. RNA was purified by phenol/chloroform extraction followed by ethanol precipitation in the presence of glycogen and was resuspended in H_2_O. For the FLAG-Xp54, injections and immunoprecipitations were performed as described before [47].

### RNA extraction from oocytes

Pools of 10 oocytes were lysed in 200 μl of TNES buffer (0.1 M Tris, pH 7.5, 0.3 M NaCl, 5 mM EDTA, 2% SDS) with the addition of 200 μg/ml proteinase K. Lysates were cleared by spinning at 12 krpm for 10 min. and incubated at 50°C for 30 min. RNA was extracted twice with acid phenol (Ambion) and once with chloroform, and then precipitated by the addition of 3 volumes of ethanol and incubation at -20°C overnight.

### Real-time quantitative RT-PCR

Two μg of total RNA in 11 μl of aqueous solution was denatured for 10 minutes at 65°C. The tubes were cooled on ice. 8 μl of master mix containing 1μl 10mM dNTPs, 1μl of each 10 mM primer (3 per reaction) and 4 μl of AMV 5x buffer (Promega) was added to each sample. The mixes were incubated at 42°C for 5 minutes. 1 μl of AMV-RT (Promega) was added to each RT+ sample and the samples were incubated at 42°C for 90 minutes. Finally, the reactions were denatured at 65°C for 10 minutes. The volume of the reaction was increased to 100 μl. 2 μl of each reaction were used for the quantitative PCR reaction using Sybr Green JumpStart Taq ReadyMix (Sigma) and 0.5 mM primers. The reaction was performed on a Rotor-Gene 6000 (Corbett), using primer pairs specified in the S1 Table.

For small RNA quantitation in oocytes and CPEB RNA-IP, miR-15b and -16a were amplified using q-RTPCR as previously described [48], with the exception that total RNA extracted from staged oocytes was used in the assay rather than a small RNA fraction. To ensure that the RT reactions were biochemically as similar as possible, 150–300 ng RNA extracted from input oocyte lysate or the indicated immunoprecipitates (irrespective of the number of oocytes the RNA represented) were polyadenylated and reverse-transcribed primed with GCGAGCACAGAATTAATACGACTCACTATAGGTTTTTTTTTTTTVN. To ensure accuracy and specificity of measurement, and in compliance with miQE guidelines [49], the following controls were included alongside experimental samples: 2-fold lower and higher dilutions of the input RNA to demonstrate a dynamic response of the RT; a serial 5-fold dilution curve of a single RT reaction for determination of relative quantities OR a serial dilution of synthetic miRNA mimics (Qiagen) for determination of absolute quantities; a PAP-free reaction (PAP-) to insure that small RNA amplification arose from non-adenylated RNA species; an RT-free reaction (RT-) to account for RNA-independent non-specific amplification; template-free polyadenylation (PAP0), template-free RT (RT0) and template-free PCR (NTC) reactions to eliminate reagent contamination and dominant primer dimers. Real-time PCR using a universal reverse primer and a specific forward primer was carried out in triplicate, and end products were analyzed by an automated thermal dissociation curve to assure a single amplified product. For quantitation in RNA-IPs, quantity (arbitrary units; a.u.) was derived using the included reference dilution series. Outlying triplicates were manually removed, averages calculated, and the mean background signal obtained in PAP-, RT-, PAP0 and RT0 negative controls (typically <10% of sample signal) was subtracted from the sample averages. The resulting quantity was then divided by the number of oocytes represented by the RNA (a.u./oocyte). Enrichment was calculated by dividing each a.u./oocyte value obtained for immunoprecipitates by the value from the matched input RNA.

Primer sequences for miRNAs and small RNA controls are listed in the S1 Table. The maternal *Xenopus laevis* piRNA (piRNA-XL-MT3744) was described in [24].

### Polyadenylation assays

8–10 microinjected oocytes/eggs (4000 cpm/oocyte) were resuspended in 20–50 μl per oocyte TNES (0.1 M Tris-HCl pH 7.5, 0.3 M NaCl, 5 mM EDTA, 2%SDS) with 1.2 mg/ml proteinase K. Crushed oocytes were incubated with intermittent vortexing at 50°C for 30 min, and the supernatant was phenol/chloroform extracted before EtOH precipitation. The extracted RNA was resuspended in 10 μl formamide dye per oocyte and analysed in sequencing type gels. 5% acrylamide gels were used for long (~200 nt) and 6% acrylamide for short (~90 nt) RNAs. The size of RNAs was estimated by comparison with øX174 *Hae* III cut DNA [^32^P]-labelled molecular weight markers.

cDNAs for polyadenylation assays were synthesized using RNA ligation-coupled PCR as described previously [50]. Gene specific primers used: cyclin E1 GAATCTGGCATGAGTGTTG; cyclin B5 GGTCTATGAACAAAATGCCTC; cyclin B1 GGAGATCTTGTTGGCACCATGTGCTTC. PCR products were resolved on 3% agarose gels which were stained with ethidium bromide.

## Results

### Cyclin E1 expression is regulated during oogenesis and oocyte maturation

Cyclin E1 plays a critical role in the final stages of meiosis II following hormone stimulation of the oocyte [40, 41]. Early studies suggested that in *Xenopus* oocytes cyclin E1 is present at very low levels, which increase prior to meiosis II. The protein accumulates in the egg after GVBD in a phosphorylated, inactive form [37]. Two alternatively spliced forms of cyclin E1 mRNA, with the same open reading frame, were cloned from a *Xenopus* unfertilized egg cDNA library, and cyclin E1b (hereafter simply cyclin E1) mRNA was shown by Northern blot analysis to be the major cyclin E1 transcript in oocytes, eggs and early stage embryos [38].

**Fig 1:**
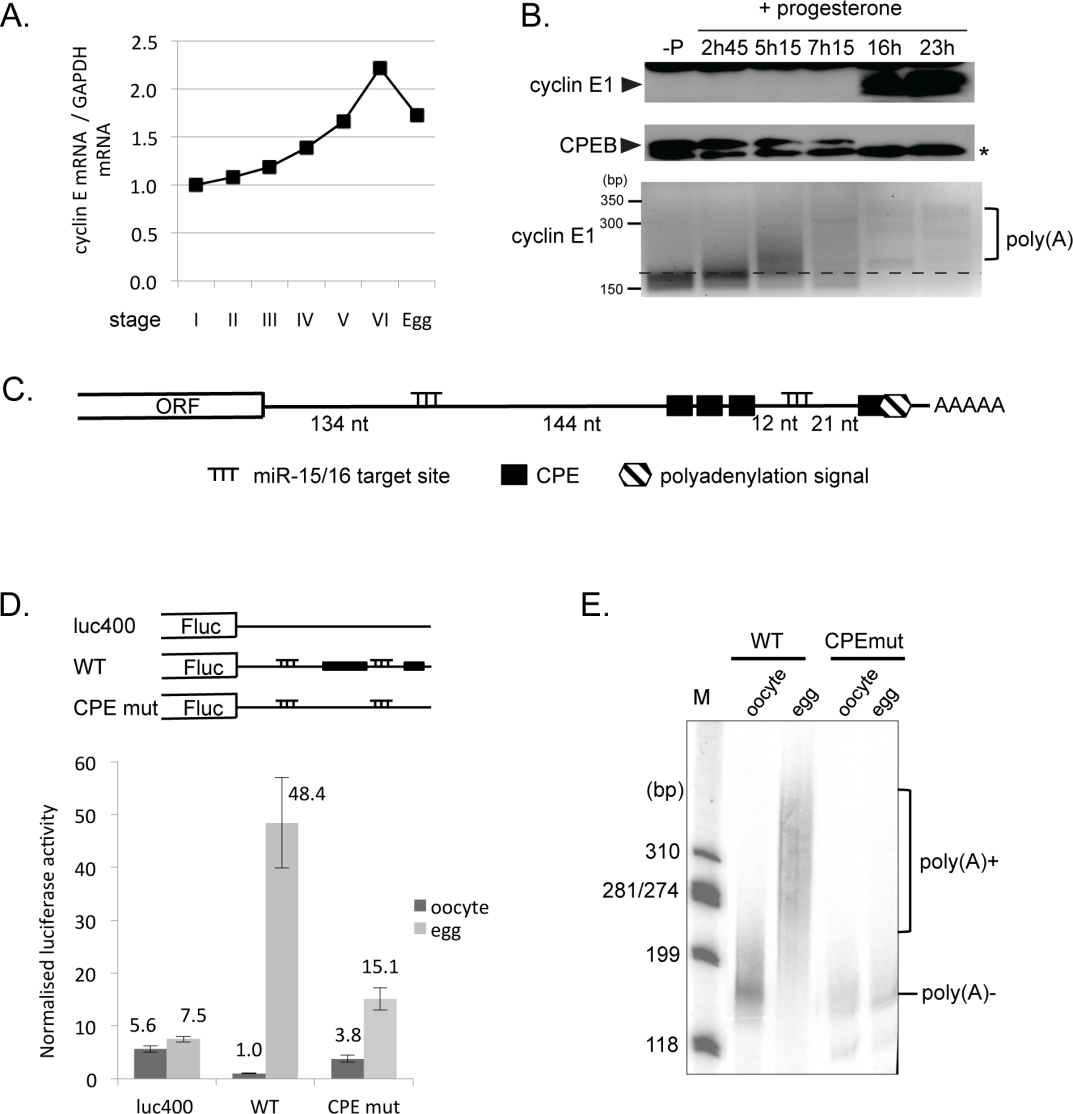
Translation of cyclin E1 during oocyte maturation is CPE-dependent. **A.** Cyclin E1 mRNA is present throughout oogenesis. RT-qPCR was carried out on total RNA extracted from staged oocytes and eggs. The graph represents the relative amount of cyclin E1 mRNA compared to GAPDH mRNA, with values of each transcript set to 1 for stage I. Representative of 5 progesterone maturation experiments. **B**. Cyclin E1 protein is not expressed until late in oocyte maturation following the polyadenylation of its mRNA. Stage VI oocytes were incubated with progesterone for the indicated times and the corresponding lysates were analysed by Western blot using cyclin E1 and CPEB antibodies (upper panels). RNA extracted from these oocytes was subjected to RNA-ligation-coupled PCR poly(A) analysis (lower panel). *—non-specific band. **C.** The cyclin E1 3’UTR contains a cluster of three CPE sequences and an additional one overlapping the hexanucleotide as well as two putative miR-15/16 target sites as indicated. Not to scale. **D**. CPE sequences repress translation in immature oocytes, and activate translation in eggs. Schematic representation of reporters used. Firefly luciferase reporters containing a control 3’UTR (luc400), the wild-type cyclin E1 3’UTR (WT) or the 3’UTR with mutations in the CPEs (CPE mut) were injected into stage VI oocytes. *Renilla* luciferase reporter mRNA was co-injected as an internal control. Oocytes were incubated for 24h with or without progesterone and both sets were assayed for luciferase expression. Firefly luciferase levels are expressed as a ratio to *Renilla* internal control. **E**. CPE sequences in the cyclin E1 3’UTR direct polyadenylation during oocyte maturation. Radiolabelled RNAs representing the WT 3’-terminal 180 nt of the cyclin E1 3’UTR and the same fragment with mutated CPE sequences were injected into oocytes, and maturation was induced by progesterone. RNA extracted from untreated oocytes and progesterone-matured eggs was analysed by denaturing gel electrophoresis and autoradiography.

We systematically assessed cyclin E1 mRNA and protein levels during oogenesis and meiotic maturation. Using RT-qPCR we show that the mRNA levels of *Xenopus* cyclin E1 double from stage I to VI of oogenesis relative to GAPDH mRNA, and then fall slightly after maturation (Fig 1A). In contrast, cyclin E1 protein is absent from oocytes, and becomes detectable only late in maturation. Its expression is seen subsequent to GVBD, which occurred around 7 hours after progesterone addition in this experiment, and after CPEB has been degraded (Fig 1B, upper two panels). Hence, translation of stored maternal cyclin E mRNA is activated in meiotically-maturing eggs. Next we assessed whether this control correlated with cytoplasmic polyadenylation. RNA analysed from the same maturation time course revealed that activation of cyclin E synthesis was preceded by polyadenylation of its mRNA (Fig 1B lower panel). Examination of the 3’UTR of *Xenopus* cyclin E1 mRNA revealed several putative regulatory sequences, including canonical CPE sequences, three in tandem and one that overlaps with the nuclear hexanucleotide AAUAAA, as well as one potential embryonic CPE (eCPE; >U_11_) [51], contiguous with the overlapping CPE (Fig 1C; S1 Fig).

### Cyclin E1 mRNA is translationally regulated by CPE sequences in oocytes and eggs

To determine whether the putative CPE sequences in the cyclin E1 3’UTR were functional in oocytes, we constructed reporter plasmids containing firefly luciferase followed by either the wild-type cyclin E1 3’UTR (WT), the cyclin E1 3’UTR containing mutations (UU->GG) in all four of the CPE sequences (CPE mut) or a control 3’UTR of a similar length (400 nt) not containing any known regulatory elements (luc400). The plasmids were linearised, *in vitro* transcribed as capped but non-adenylated mRNAs and co-injected into oocytes together with an internal control *Renilla* luciferase mRNA. RNA degradation is compromised in immature oocytes, so such RNA is stable throughout the time of the experiment (see Introduction). Following an overnight incubation in the presence or absence of progesterone, reporter levels were assessed. These revealed that the cyclin E1 3’UTR confers about 6-fold repression in the oocyte compared to the control 3’UTR, and also that CPE sequences present in the cyclin E1 3’UTR are required for this repression, as mutation of these sequences resulted in a considerable albeit incomplete derepression of the reporter constructs (Fig 1D, oocyte).

Furthermore, maturation of oocytes into eggs resulted in a very robust activation (>40-fold) of translation of the reporter construct containing the WT 3’UTR (Fig 1D, egg). This effect of the WT 3’UTR can be viewed as comprised of the ~6-fold baseline repression of translation in oocytes coupled with a ~7-fold activation of translation upon maturation. Both effects are largely, but not completely, dependent upon intact CPEs. Moreover, as shown previously for cyclin B1 mRNA [52], mutation of the nearby nuclear hexanucleotide AAUAAA to AAGAAA also abrogates translational activation of cyclin E1 mRNA, even in the presence of all CPE elements (data not shown). Thus cyclin E1 mRNA is translationally activated in a manner requiring CPE elements and the nuclear hexanucleotide, hallmarks of cytoplasmic polyadenylation.

### Cyclin E1 mRNA is polyadenylated in the cytoplasm *via* CPE elements during meiotic maturation

Polyadenylation of cyclin E1 mRNA was examined by microinjecting ^32^P-labelled 3’UTR transcripts into the cytoplasm of oocytes, some of which were matured with progesterone. After an overnight incubation, total RNA from oocytes and eggs was isolated and analysed by denaturing polyacrylamide gel electrophoresis, showing that the wild type cyclin E1 3’UTR RNA was extended by some 170 nt following progesterone-stimulated maturation, but that no extension was seen with cyclin E1 3’UTR RNA mutated in the CPE sequences (Fig 1E). Control experiments using oligo(dT)-directed RNase H cleavage confirmed that the extension was indeed due to polyadenylation (data not shown). We also showed, using immunoprecipitation of lysate proteins UV-crosslinked with wild type and mutant 3’UTR RNAs, that CPEB binding to cyclin E1 3’UTR requires the CPE elements (data not shown). Altogether we conclude that cyclin E1 levels are dictated by 3’UTR CPE elements, which mediate repression in oocyte and translational activation following CPEB-driven cytoplasmic polyadenylation during oocyte maturation.

### Cyclin E1 mRNA is a miR-15/16 target in the oocyte

**Fig 2:**
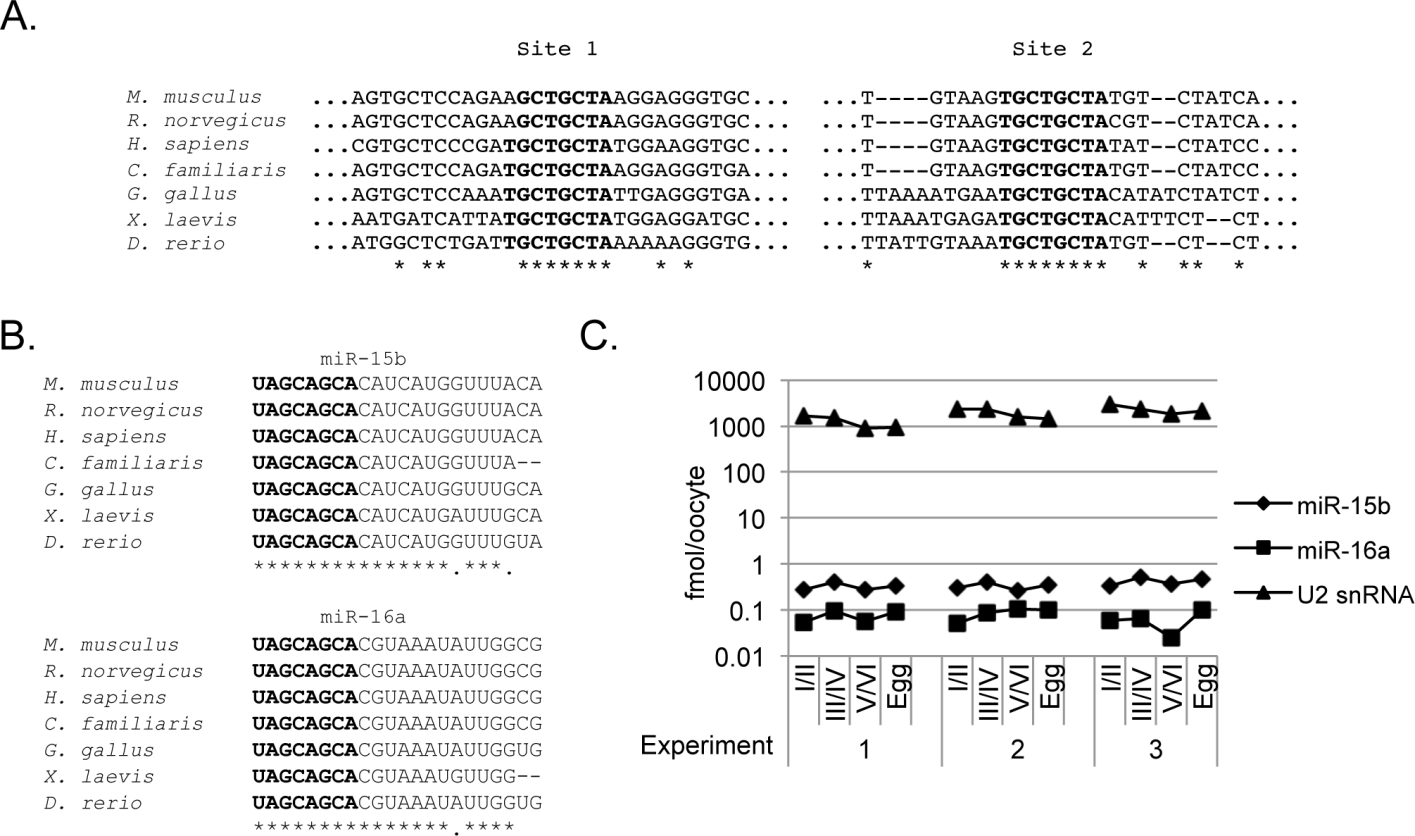
Mature forms of miR-15 and miR-16 are present in *Xenopus* oocytes. **A**. Alignment of putative miR-15/16 seed binding sites across vertebrate cyclin E1 3’UTRs. **B**. Alignment of vertebrate miR-15b and miR-16a reveals nearly perfect conservation of the two miRNAs, with the seed sequence highlighted in bold. **C**. miR-15b and 16a levels do not undergo significant changes during oogenesis and oocyte maturation. Levels of the two microRNAs were verified by qPCR alongside the control U2 snRNA. The graph represents absolute quantities throughout oogenesis in three independent biological samples.

Further examination of the *Xenopus* cyclin E1 3’UTR revealed, aside from the polyadenylation elements, the presence of two miR-15/16 target sites characterized previously in mammalian cyclin E1 mRNAs [33–36]. These sites are conserved in vertebrates, including zebrafish and chicken (Figs 1C and 2A; S1 Fig), similarly to miR-15b and miR-16a. Overall, the 3’UTRs show no stretch of conservation apart from the miRNA sites. Indeed, full seed sequence complementarity is maintained among vertebrate species and to a large extent within the remainder of the mature miRNA (Fig 2B). Previously, we identified miRNAs 15a and 16b in *Xenopus tropicalis* oocytes [22], in line with similar systematic deep sequencing studies in *Xenopus laevis* and *tropicalis* oocytes and eggs [24]. Here, quantitation of these microRNAs by real-time RT-PCR analysis from staged *Xenopus laevis* oocytes revealed that mature species of both miRNAs are detectable throughout oogenesis, with the levels of miR-15b significantly higher (0.1–1 fmol per oocyte) than that of miR-16a (0.01–0.05 fmol per oocyte) (Fig 2B and 2C) as assessed by quantitative PCR using miRNA mimics as reference.

**Fig 3:**
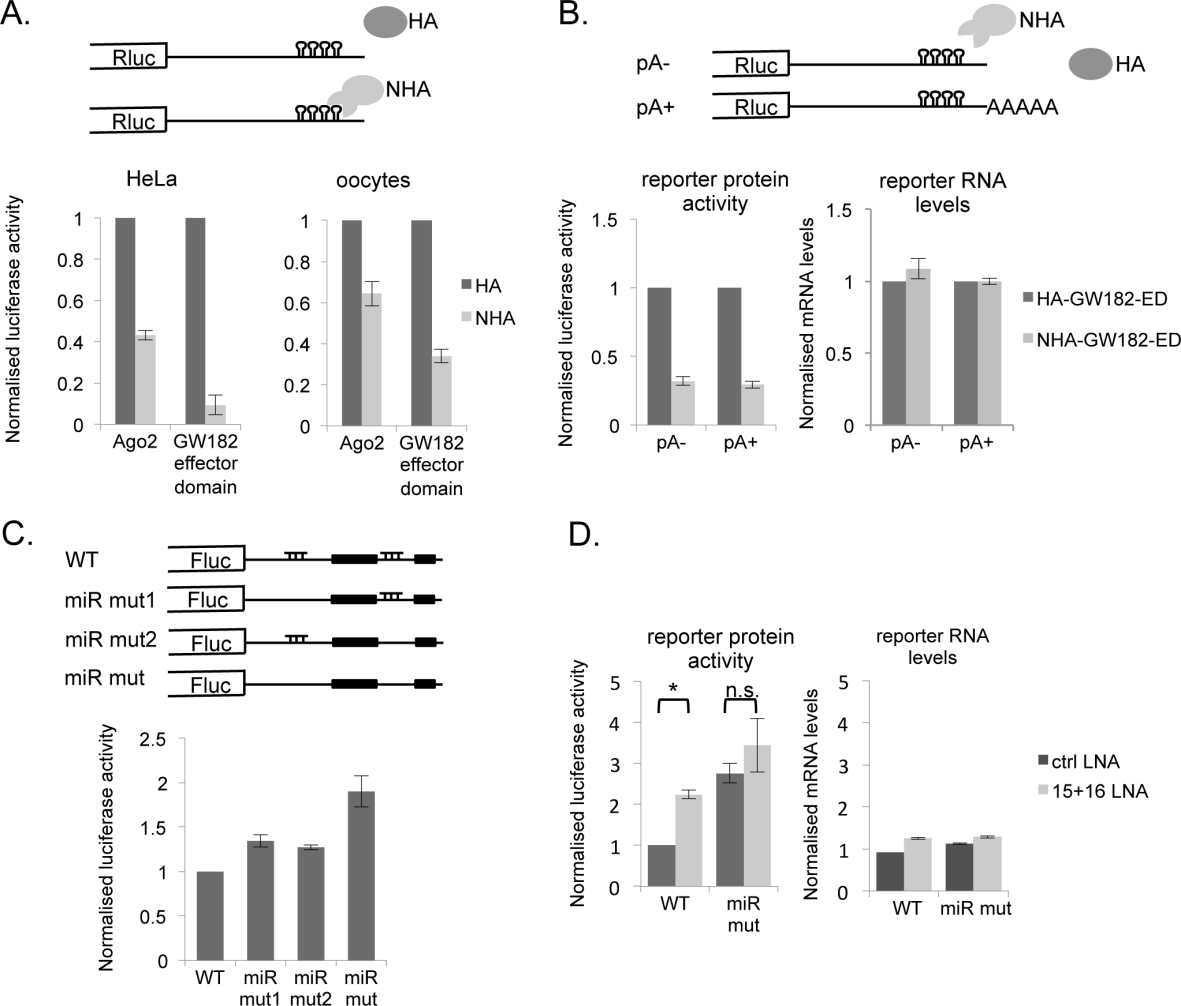
The miRISC as well as the putative miR-15/16 target sites in the cyclin E1 3’UTR are functional. **A.** Tethering of either Ago2 or the effector domain of GW182 represses translation of a reporter in mammalian cells as well as in *Xenopus* oocytes. Schematic representation of reporters used. mRNAs encoding lambda-N peptide with an HA-tag or an HA-tag only, fused to GW182 effector domain or Ago2 were either injected into oocytes for 24 h prior to injection of *Renilla* luciferase reporter mRNA containing 3’UTR Box B sites (Rluc-BoxB) or transfected into HeLa cells 24 h prior to transfection of plasmids encoding the reporter genes. An mRNA/plasmid encoding Firefly luciferase was co-injected/co-transfected as an internal control. After a 6 h incubation, cells were harvested and reporter protein expression was assessed. The experiment was repeated 3 times with similar results. **B**. Repression of a reporter mRNA tethered to the effector domain of GW182 is independent of a poly(A) tail. Schematic representation of reporters used. The experiment was performed in *Xenopus* oocytes as in A, using either non-adenylated (pA-) or *in vitro* polyadenylated (pA+) RNA. RNA was assessed from a pool of 50 injected oocytes. **C.** The two miR-15/16 sites co-operate in repressing translation of the cyclin E1 3’UTR. Luciferase reporters containing the wild-type cyclin E1 3’UTR (WT), mutations in the first miR target site (miR mut1), the second target site (miR mut2) or both (miR mut) were injected into stage VI oocytes. *Renilla* luciferase mRNA was co-injected as an internal control. Firefly luciferase levels are expressed as a ratio to *Renilla* internal control. The graph displays the results for a representative experiment. **D**. The miR-15/16 target sites are active in a degradation-independent manner. Luciferase reporters containing the wild-type cyclin E1 3’UTR (WT) or the cyclin E1 miR mut 3’UTR were injected into oocytes (see C for further details), in the presence of co-injected control or miR-15/16 LNAs, as indicated. * Student t-test P<0.01. RNA extracted from injected oocytes was subjected to reverse transcription and quantitative real-time PCR to assess RNA levels, which are expressed as a ratio of firefly to *Renilla* luciferase. The graph displays the results for a representative experiment.

To assess whether the RISC complex is functional in the *Xenopus* oocyte, we utilised λN/box B constructs previously used in tethering assays in mammalian cell lines [53, 54]. In this assay, a λN-tagged effector protein of the miRNA machinery (Ago2 or TNRC6) is tethered *via* five Box B sites to the 3’UTR of a reporter mRNA, mimicking the action of a microRNA (see Fig 3A). It is particularly appropriate in cells such as the non-dividing oocyte from which proteins cannot be readily depleted (reviewed in [45]). We therefore compared the ability of Ago2 and GW182 to influence tethered *Renilla* luciferase reporter mRNA, alongside a control Firefly luciferase mRNA, in transfected HeLa cells and in microinjected *Xenopus* oocytes. In the case of GW182, we tethered the Δ1370 C-terminal effector domain of human TNRC6C [42], as this is well-expressed in both cell types.

As previously reported in HeLa cells [42, 53, 54], λN-Ago2 and -GW182 reduce Rluc-levels, but not Fluc protein levels, relative to proteins lacking the λN peptide, with the silencing domain of GW182 being more effective than Ago2 (Fig 3A, left panel), likely due to its ability to act downstream of Ago2. Interestingly, we found that GW182 was also more effective in oocytes, compared to Ago2, though the proteins were equally well expressed (data not shown). The degree of silencing was somewhat lower in oocytes relative to HeLa cells (Fig 3A, right panel), reflecting, at least in part, that mRNA decapping is compromised in the oocyte (see Introduction). Indeed, silencing in oocytes is entirely at the translational level, as shown by the unchanging reporter mRNA levels determined by qRT-PCR (Fig 3B). Lower levels of required components [55] could also contribute to the reduced silencing in oocytes. In addition, unlike the mRNA expressed in HeLa cells, the mRNA injected into oocytes is typically capped but non-adenylated, to reflect the state of maternal mRNAs. We found, however, that using poly(A) polymerase to add a poly(A) tail to the tethering mRNAs did not affect the extent of silencing in oocytes (Fig 3B).

Next, to test whether the miR-15/16 target sites were active in the oocyte, we created firefly luciferase-cyclin E1 3’UTR constructs containing single mutations in each of the sites (miR mut1 and miR mut2, respectively) and mutations in both sites (miR mut), and compared them with the reporter bearing the wild type cyclin E1 3’UTR. Reporter mRNAs and control *Renilla* luciferase mRNA were injected into immature stage VI oocytes as before, and their expression assayed after 6 h. These experiments showed that the miRNA target sites contribute to the translational repression of cyclin E1 mRNA, and that the combined effect of mutating both sites is additive, resulting in approximately 1.5–2 fold increase in translation (Fig 3C).

To validate further the cyclin E1 mRNA microRNA target sites, we pre-injected stage VI oocytes with either a control LNA oligonucleotide or a mix of anti-miR-15 and -miR-16 inhibitor LNAs. Luciferase activity levels were assayed after subsequent reporter injection and incubation. These revealed that mutation of the miRNA target sites resulted in a 2-fold de-repression of the reporter in the presence of the control non-target LNA oligo (Fig 3D, protein level panel). Importantly, the pre-injection of specific anti-miR-15 and–miR-16 LNA inhibitors resulted in the de-repression of the WT reporter, while the mutant mRNA was not significantly affected. Furthermore, we demonstrated that RNA levels were unchanged (Fig 3D, RNA levels panel). In conclusion, the effect of mutating the miR-15/16 sites and/or inhibiting endogenous miR-15/16 using LNAs occurs largely on the translational level.

### CPEB complexes are associated with miRISC

The experiments described above established that introduction of miR site mutations in the context of the WT cyclin E1 3’UTR (i.e. in the presence of CPEs and therefore presumably in association with CPEB) resulted in a partial derepression of the reporter mRNA (Fig 3). Previously, we showed that CPEB is found in a very large RNP complex in oocytes, and mRNAs and partner proteins, including cyclin B1 mRNA and Xp54/DDX6 RNA helicase, can be readily co-immunoprecipitated with CPEB antibodies [5].

**Fig 4:**
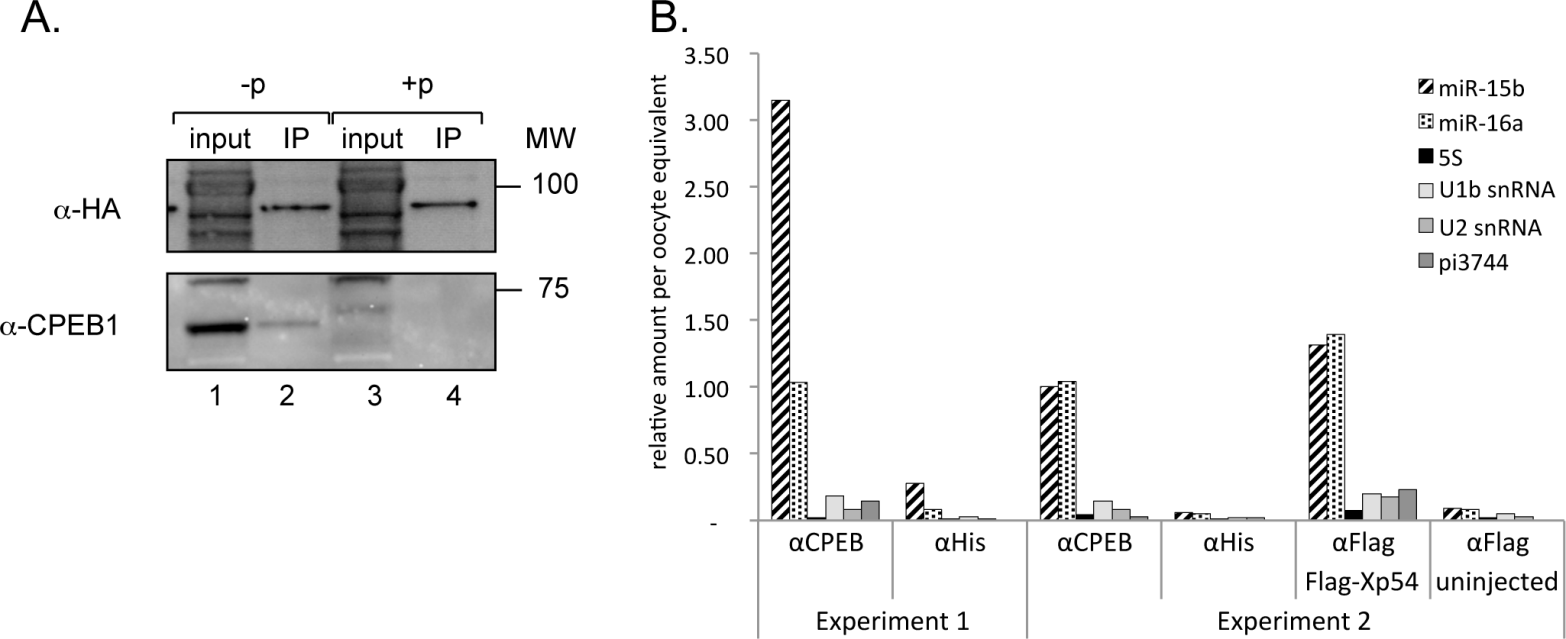
CPEB complexes and miRISC cooperate in the regulation of translational activation of cyclin E1 during oocyte maturation. **A.** Components of the RISC complex can associate with the translational silencing complex in immature oocytes (-p). Oocytes were injected with HA-Ago2 mRNA, and the resulting lysates were subjected to immunoprecipitation with anti-HA antibodies. Input represents 5% of the immunoprecipitated fractions. Western blotting was performed with anti-HA or -CPEB1 antibodies and visualised by ECL.–p–no progesterone, immature oocytes; +p–oocytes matured with progesterone. **B**. miRNAs co-immunoprecipitate with CPEB complexes. Lysates from uninjected oocytes or oocytes expressing a FLAG-tagged Xp54 were used for immunoprecipitation using a monoclonal anti-CPEB antibody, an isotype-matched control (His), or in the case of FLAG-Xp54 expressing oocytes and uninjected control (“-“), an anti-FLAG antibody (FLAG). RNA was isolated and real-time RT-PCR performed for the miRNAs and small RNAs indicated. Relative quantities were normalised per oocyte input. For plotting purposes all enrichment values were scaled to the enrichment of miR-15 in the CPEB immunoprecipitate of Experiment 2.

To address whether the RISC complex interacts with CPEB, indicating a potential combined mode of translational control, we injected mRNAs encoding HA-tagged *Xenopus tropicalis* Ago2 protein into stage VI oocytes, subsequently incubated with and without progesterone. Immunoprecipitation using an anti-HA antibody revealed that endogenous CPEB co-immunoprecipitates with HA-Ago2 in oocytes (but not in eggs when CPEB is degraded), suggesting an interaction between the miRNA machinery and the CPEB RNP (Fig 4A). We were able to demonstrate that this association is RNA-independent in immunoprecipitations performed in the presence of RNAse (S2 Fig).

Next, we assessed the presence of miRNAs in association with the CPEB complex. Immunoprecipitation was performed with stage VI oocyte lysates prepared from 2 different frogs, using a monoclonal anti-CPEB antibody [5] and an unrelated isotype-matched control antibody (anti-His). Bound RNA was extracted and analysed by real time RT-PCR to reveal that both miR-15b and -16a, but not a piRNA (pi3744; [24]) of similar length and abundance as miR-15b, are specifically co-precipitated with CPEB. Moreover, the abundant 5S rRNA, U1b or U2 snRNAs were also not co-precipitated with CPEB. A similar specific enrichment was measured in anti-FLAG immunoprecipitates of oocytes injected with mRNA encoding FLAG-tagged Xp54/DDX6, CPEB’s interacting partner [56], but not of uninjected oocytes (Fig 4B). Altogether we conclude that CPEB interacts with Ago2 and microRNAs. The coordinated regulation resulting from co-residence of miRISC and other complexes involved in translational regulation, such as the CPEB RNP complex, is an intriguing concept, adding an additional level to the complexity of post-transcriptional regulation [57].

### Inhibition of miR-15/16 causes premature polyadenylation of cyclin E1 mRNA and acceleration of meiotic maturation

**Fig 5:**
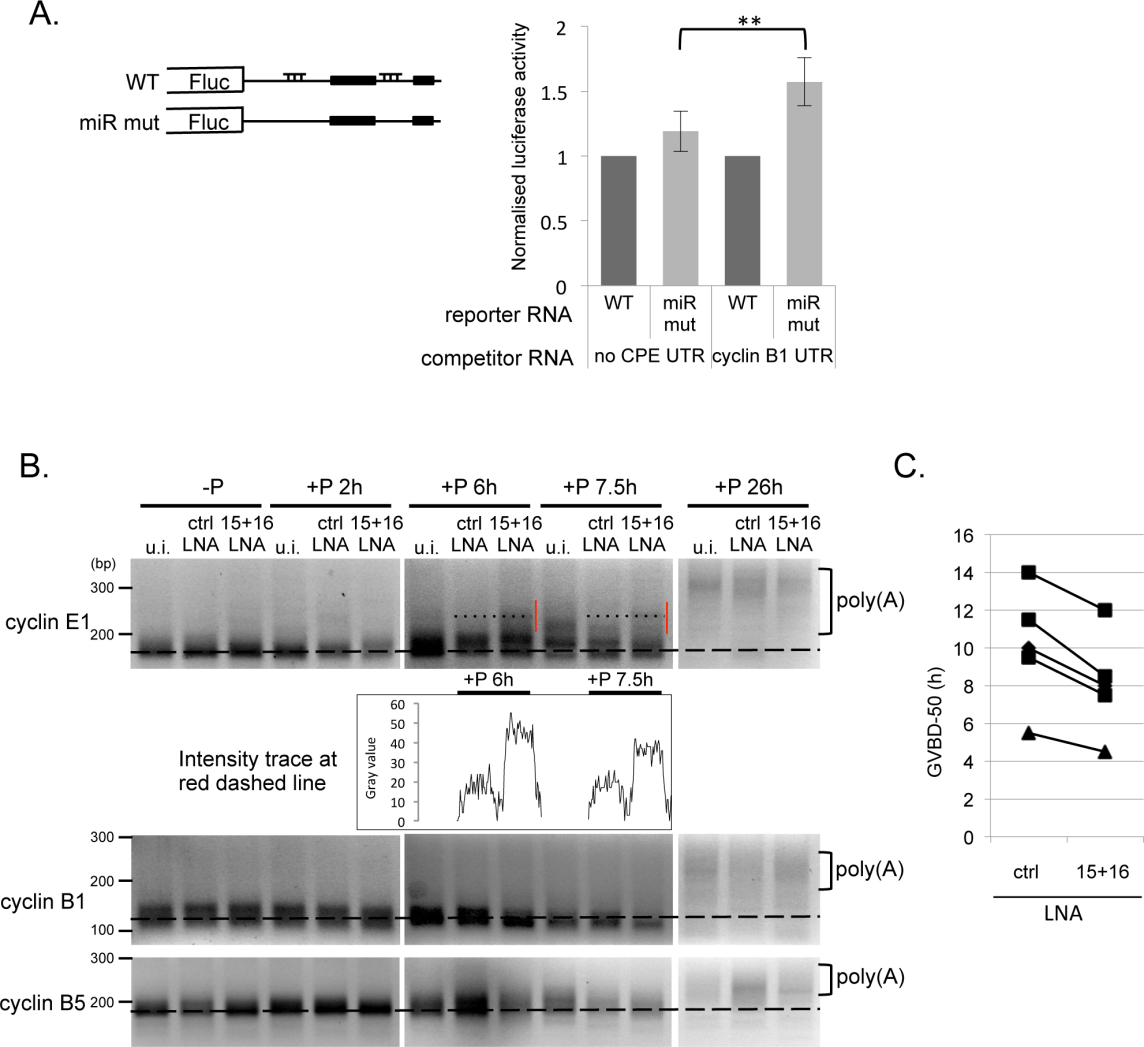
Inhibition of miR-15/16 causes premature polyadenylation of cyclin E1 mRNA and acceleration of meiotic maturation. **A.** Injection of a molar excess of CPE-containing RNA competitor enhances the effect of miR site mutations. 500 fmol of either a control 85 nt RNA not containing any CPE sequences (no CPE UTR) or a 65 nt 3’–terminal sequence of the cyclin B1 3’UTR (cyclin B1 UTR) were injected into stage VI oocytes. After an overnight incubation, oocytes were re-injected with either the WT or miR mut Firefly reporter constructs and the *Renilla* control RNA. The oocytes were lysed and assayed for luciferase after 6 h. The graph represents and average of 3 experiments with the WT reporter normalised to 1 in each case. (** P<0.01, 2-tailed paired t-test). **B**. Uninjected stage VI oocytes (u.i) or oocytes injected with control LNA (ctrl LNA) or a mixture of anti-miR-15 and anti-miR-16 LNAs (15+16 LNA) were incubated overnight, following which they were stimulated with progesterone. Samples were taken at indicated times. Extracted RNA was subjected to RNA-ligation coupled PCR polyadenylation analysis using primers for indicated mRNAs. Vertical red line indicates early polyadenylation in 15+16 LNA injected sample. Panel shows a representative experiment of 4. Graph underneath cyclin E panel depicts ImageJ profile plot taken along the dotted line. **C**. 100–150 stage VI oocytes were injected with either control LNA (ctrl LNA) or anti-miR-15 and anti-miR-16 LNAs (15+16 LNA) and incubated overnight and then treated with progesterone. GVBD was scored by the appearance of a white spot on the animal pole of the oocyte. The time required for 50% of the oocytes to achieve GVBD (GVBD-50) is plotted for 5 independent experiments.

The critical aspect of CPEB activity is the precisely-timed relief of repression and subsequent activation of translation of its target mRNAs during oocyte maturation, prior to its proteolysis. A previous study developed a method to show these two functions in greater temporal resolution, by the injection of short CPE-containing RNA that acts as a competitor for CPEB protein, which first triggers the derepression of endogenous CPE-containing mRNAs followed later by polyadenylation-mediated activation [2]. We injected the CPE-containing fragment of the cyclin B1 3’UTR or a control RNA of similar length. Following an overnight incubation, oocytes were injected with WT or miR mut reporter constructs and incubated as for other reporter experiments. As expected, the translation of reporter mRNA with CPE elements was derepressed (approximately 3-4-fold) by prior injection of short CPE RNA relative to control RNA, demonstrating that competing CPEB off the target mRNA was effectively mimicking early maturation events. Interestingly, we observed a statistically significant increase in the extent of derepression of the miR mut construct in oocytes with limiting CPEB levels (pre-injected with CPE-RNA) compared to control oocytes (pre-injected with control RNA) (Fig 5A). These results suggest that while in immature oocytes, miRISC requires CPEB to act upon the cyclin E1 mRNA, in an environment in which CPEB is being competed off the CPE-containing mRNAs, miRISC reinforces the interaction of CPEB with the same mRNA. It is tempting to speculate that the co-occupancy of the two complexes on the mRNA serves to stabilise each other when in close proximity.

To test this possibility, we examined the role of this regulation in the activation of cyclin E1 mRNA during oocyte maturation. Since we have shown that this activation is preceded and driven by the polyadenylation of the mRNA (Fig 1), we analysed the poly(A) status of endogenous cyclin E1 mRNA during a time course following injection of specific LNA oligos inhibitory to miR-15 and -16, or control LNAs (validated in Fig 3D), or in uninjected oocytes (Fig 5B). Oocytes were incubated overnight, and then subjected to progesterone treatment. Samples were taken at indicated time points and extracted RNA was subjected to the RNA ligation-linked poly(A) assay [50]. We observed that in oocytes injected with the anti-miR-15/16 LNAs, an initial limited polyadenylation of cyclin E1 mRNA was observed at 6 h, which for the uninjected and control LNA injected oocytes did not occur until 7.5 h. In contrast, there was no change in the timing of polyadenylation of cyclin B1 or B5 mRNAs, which have no miR-15/16 sites in their 3’UTRs, suggesting that the inhibition of miR-15/16 specifically prematurely activates cyclin E1 mRNA polyadenylation.

Finally, determining the GVBD timing during a maturation time course in five experiments revealed that injection of anti-miR-15/16 LNAs accelerates maturation of oocytes when compared to injection of control LNA (Fig 5C), presumably as a result of the early polyadenylation and translational activation of cyclin E1 and potentially other target mRNA when miR-15/16 levels are reduced (Fig 5B).

On the basis of the reporter mRNA analyses and the oocyte experiments, we conclude that miR-15/16 and CPEB co-regulate cyclin E1 mRNA. Moreover, inhibition of miR-15/16 in the immature oocyte results in accelerated meiotic maturation reinforcing the notion of these miRNAs playing major roles as cell cycle regulators. To our knowledge this is the first demonstration of microRNAs regulating the timing of mRNA polyadenylation.

## Discussion

We report that the translation of cyclin E1 mRNA is tightly regulated in the *Xenopus* oocyte and egg by its 3’UTR CPE elements, and two neighbouring conserved miR-15/16 sites. The CPE elements and the miRNA binding sites repress translation in the oocyte and during meiotic maturation, and CPE elements promote cytoplasmic polyadenylation and translational activation in the egg. Tight regulation of cyclin E1 levels presumably reflects its important roles in cell cycle progression as it is required for metaphase II arrest in the egg as well as S-phase initiation in the early embryo [40, 41].

Reflecting our interest in the cytoplasmic control of cyclin E1 mRNA poly(A) tail length and translation, our analysis was based on the microinjection of mRNAs (and LNA antagomirs) into the cytoplasm of defolliculated stage VI oocytes, which can meiotically mature in response to progesterone. It is interesting to note that a study using folliculated stage IV-VI *Xenopus* oocytes and relying on nuclear co-injection of reporter mRNA and microRNA concluded that microRNAs activate translation [58].

Dual regulation of translation by CPE elements is a well-known mechanism to control protein levels during oogenesis and meiotic maturation, and the extensively characterised examples include members of the *Xenopus* cyclin B mRNAs [11]. Indeed, the number and relative location of the CPE elements in the cyclin E1 3’UTR conform very well to the rules obtained in this landmark paper on the combinatorial code for cytoplasmic polyadenylation elements. For an mRNA to be repressed in the oocyte requires a cluster of at least two CPEs, optimally separated by 10–12 nt, while cytoplasmic polyadenylation in the maturing egg following progesterone stimulation requires at least one CPE located less than 100 nucleotides from the nuclear hexanucleotide signal, and late polyadenylation (at or after GVBD) is directed by a CPE element overlapping the hexanucleotide. As previously reported, the putative eCPE (>U_11_, [51], the hexanucleotide and an upstream AU-rich element (very likely corresponding to our cluster of three CPEs) promote polyadenylation of cyclin E1 mRNA later in development, during *Xenopus* embryogenesis [59].

Comparison of cyclin E1 3’UTRs in *Xenopus*, zebrafish, mouse and man (S1 Fig) revealed that consensus CPEs (U_4_A_1-2_U) characterised experimentally here in *Xenopus* are not well conserved in mammals. Indeed, human and mouse cyclin E1 appear to have only the CPE element that overlaps the hexanucleotide, though possibly non-consensus CPEs, UA-rich elements comprising at least four U bases, may operate here. Alternatively, the rules obtained for maturing *Xenopus* oocytes [11] may not readily transfer to other organisms. Indeed it has been noted that in the case of the cyclin B1 3’UTR, zebrafish diverges from *Xenopus*, with the positions and sequences of the functionally defined CPEs being poorly conserved [60]. In the case of the cyclin E1 3’UTR, the upstream CPE elements are present in zebrafish, but the overlapping one is absent, though an additional potential *D*. *rerio* CPE element is found downstream of the hexanucleotide (S1 Fig), in a similar configuration to that observed in its cyclin B1 mRNA [60].

In contrast, both cyclin E1 miR-15/16 binding sites are highly conserved from frog to man, one site just upstream (~10–25 nt) of the hexanucleotide, with the second one located ~ 250 nucleotides upstream (S1 Fig). Using microinjected reporter-cyclin 3‘UTR mRNAs with wild type or mutated miRNA binding sites, we showed that each functioned to moderately repress translation in the oocyte, with an additive effect being observed when both sites were mutated; in agreement with studies in mammalian cells [33, 35, 36]. Furthermore, we showed that miRNAs repress translation in the oocyte, with no significant effects on reporter mRNA levels. Similarly, in tether function assays we noted that the effector domain of GW182 represses bound mRNA, rather than destabilising it. Moreover, no additional repression was observed when a poly(A) tail was added to the reporter mRNA prior to injection. In several studies, a poly(A) tail was noted to enhance but not be required for microRNA silencing ([61] and references therein) possibly due to enhanced decay. Indeed, the effector domain of GW182 interacts with components of the CCR4–NOT and PAN2/PAN3 deadenylase complexes as well as PABP, resulting in target mRNA deadenylation, and ultimately decay [62–64]. Interestingly, deadenylases also exert translational repression, mediated by the recruitment of DDX6 by CNOT1 [65–67].

As discussed in the Introduction, mouse maternal miRNAs do not appear to be essential for proper development, though reporter studies indicate they may play a role in regulating expression of target mRNAs, depending on the identity and/or number of microRNA-binding sites [25–28]. We provide evidence in this study of a contributing role of microRNAs in silencing a CPE-target mRNA in *Xenopus* oocytes and eggs. Such a role may be more important in *Xenopus* development as mouse maternal mRNA is degraded at the 2 cell-stage in contrast to the ~4096 cell stage during *Xenopus* embyogenesis, at the mid-blastula transition, when zygotic transcription is initiated. Interestingly, the rapid deadenylation and clearance of maternal mRNAs is mediated by a conserved microRNA, miR-430 in zebrafish and miR-427 in *Xenopus* embryos, synthesised after fertilisation [68, 69].

**Fig 6:**
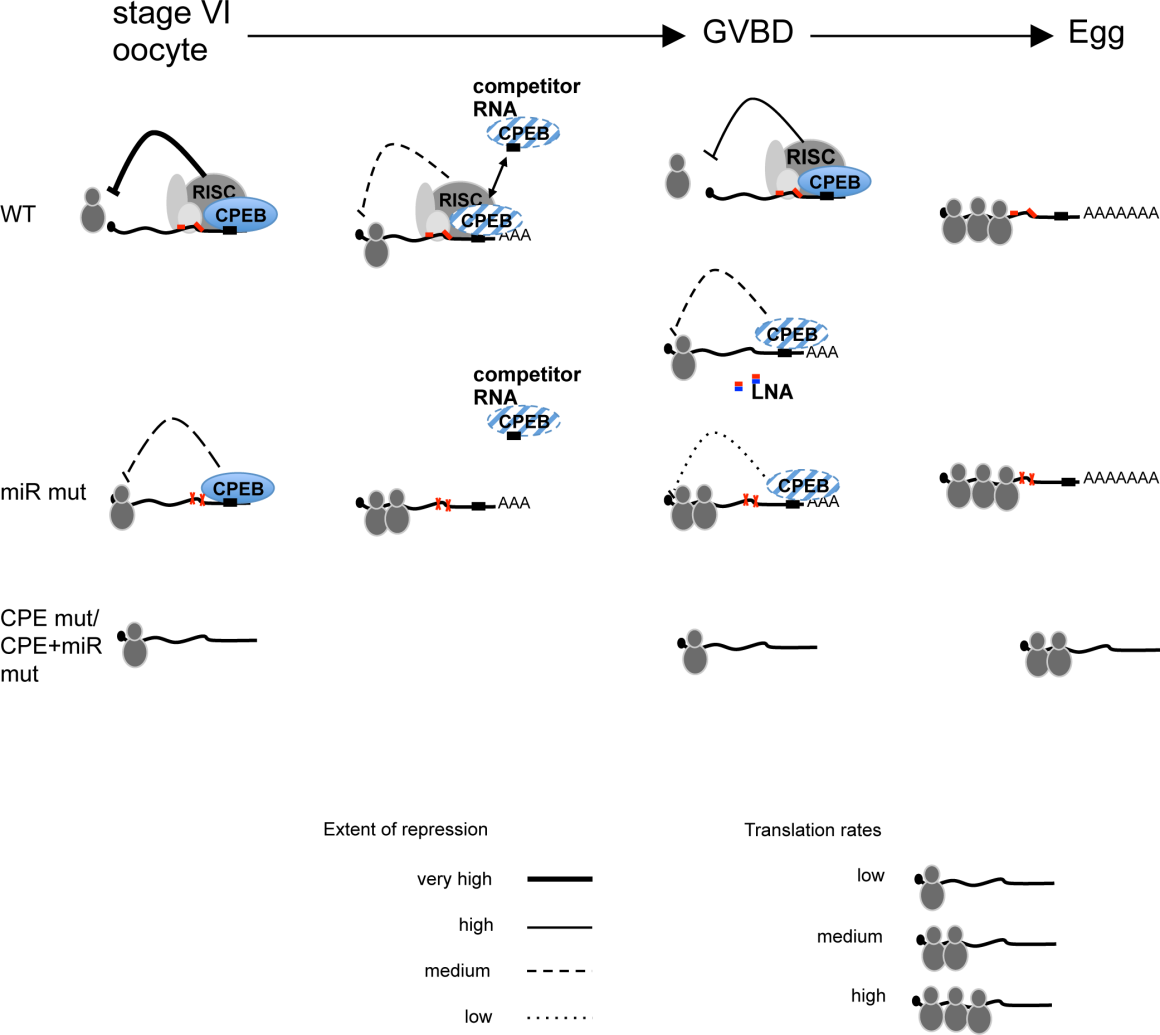
Schematic summary of results and model of action of CPE- and microRNA-binding sites in cyclin E1 mRNA during meiotic maturation. In the immature oocyte, the presence of CPE sequences on maternal mRNAs stabilises the association of miRISC with the target mRNA to cause augmented translational repression (WT vs. miR mut). In the absence of either the CPE sequence alone or both CPEs and miRNA target sites, the mRNA cannot bind the CPEB RNP nor RISC and is therefore not repressed. In the presence of RNAs competing for CPEB, miRISC acts to reinforce the interaction of the limited CPEB protein with the mRNA resulting in lower translation rates compared to the miR mut mRNA which does not bind miRISC. During GVBD, when the degradation of CPEB begins, once again, co-association of CPEB and miRISC with an mRNA stabilises both interactions and delays polyadenylation and translational activation. Mutation of the miRNA target sites or inhibition of miR-15/16 allows for early polyadenylation and activation of the transcript. In our model, we have not distinguished the functionally similar but temporally distinct roles of CPEB1 and CPEB4 [72].

The focus of our work was cyclin E1 mRNA whose expression during oogenesis and meiotic maturation is tightly regulated by 3’UTR CPE elements, and by two miR-15/16 binding sites (summarised in Fig 6). Our results provide evidence of how miRNAs may function to add an additional level of control over major regulatory mechanisms such as CPEs/CPEB. While the miRNA sites are not absolutely required for either the repression or activation of cyclin E1 mRNA, they provide kinetic fine-tuning in a process in which timing is critical. Inhibition of miR-15/16 leads to the accelerated cytoplasmic polyadenylation of the cyclin E1 mRNA. Even more strikingly, injection of anti-miR-15/16 LNAs resulted in a marked and significant acceleration of GVBD compared to control sequences suggesting additional miR-15/16 targets in addition to cyclin E1. In mammalian cells, the miR-16 family regulates cell cycle progression and proliferation by regulating multiple cell cycle genes [33, 35, 70, 71].

We propose that CPEB facilitates the binding of miRISC to the mRNA containing both regulatory sequences (miRNA target site and CPE), which exerts additional repression in the immature oocyte. The onset of maturation causes selective phosphorylation and degradation of CPEB, but miRISC, which has already been put in place, delays the polyadenylation and also potentially stabilises the association of the repressive form of CPEB on the mRNA, ultimately resulting in the late activation of the mRNA. This would be consistent with the well described activity of miRISC in promoting the deadenylation of target mRNAs.

There is mounting evidence for the existence of interplay between regulatory sequence elements within 3’UTRs. A bioinformatics study revealed that in experiments where miRNA function is inhibited, upregulated mRNAs show both positive and negative correlations for certain RNA-binding proteins (RBP) target sequences, suggesting that some regulatory elements cooperate, while others compete with miRNA target sites [73]. Interestingly, most cooperative effects, resulting in the preferential destabilisation of the mRNA, have been seen between miRNA target sites, AU-rich elements and/or CPEs [39, 74–76]. Indeed, we (this study) and others [39] have shown that these three features are all involved in regulating *Xenopus* cyclin E1 expression. Additional examples include Pumilio, an important RBP involved in control of development and the cell cycle, which also cooperate with miRNAs to regulate target mRNAs [77, 78]. Our results support the notion of such a mechanism being widespread. Moreover, we provide the first evidence of miRNAs contributing to the regulation of the precise timing of relief of translational repression. Such mechanisms could be of great importance not only in early development but also in neuronal function where localisation and timing of translational activation are critical.

## Supporting Information

S1 Fig Sequence alignment of the human, mouse, frog and zebrafish cyclin E1 3’UTRs using ClustalW2. CPE sequences are in bold and italic (overlapping CPE), the nuclear hexanucleotide is bold and blue, and the two miR-15/16 binding sites are coloured yellow. Accession numbers as follows: Hs BC035498; Mm NM_007633; Xl3.1-IMAGE:6638064.5.5; Dr X83594.
(TIF)

S2 Fig Interaction of CPEB and Ago2 in the *Xenopus* oocyte is RNA-independent. Oocytes were injected with FLAG-Ago2 mRNA, and the resulting lysates were subjected to immunoprecipitation with anti-FLAG magnetic beads in the presence of RNAse A. Input represents 10% of the immunoprecipitated fractions. Western blotting was performed with anti-FLAG or -CPEB1 antibodies. The arrow indicates the CPEB band.
(TIF)

S1 Table Sequences of primer sequences used in this study. (PDF)
