# Supplementary figures and images for: CPEB and miR-15/16 Co-Regulate Translation of Cyclin E1 mRNA during Xenopus Oocyte Maturation
Source: PLoS One. 2016 Feb 1;11(2):e0146792. doi: 10.1371/journal.pone.0146792 (PMC4734764; doi:10.1371/journal.pone.0146792)

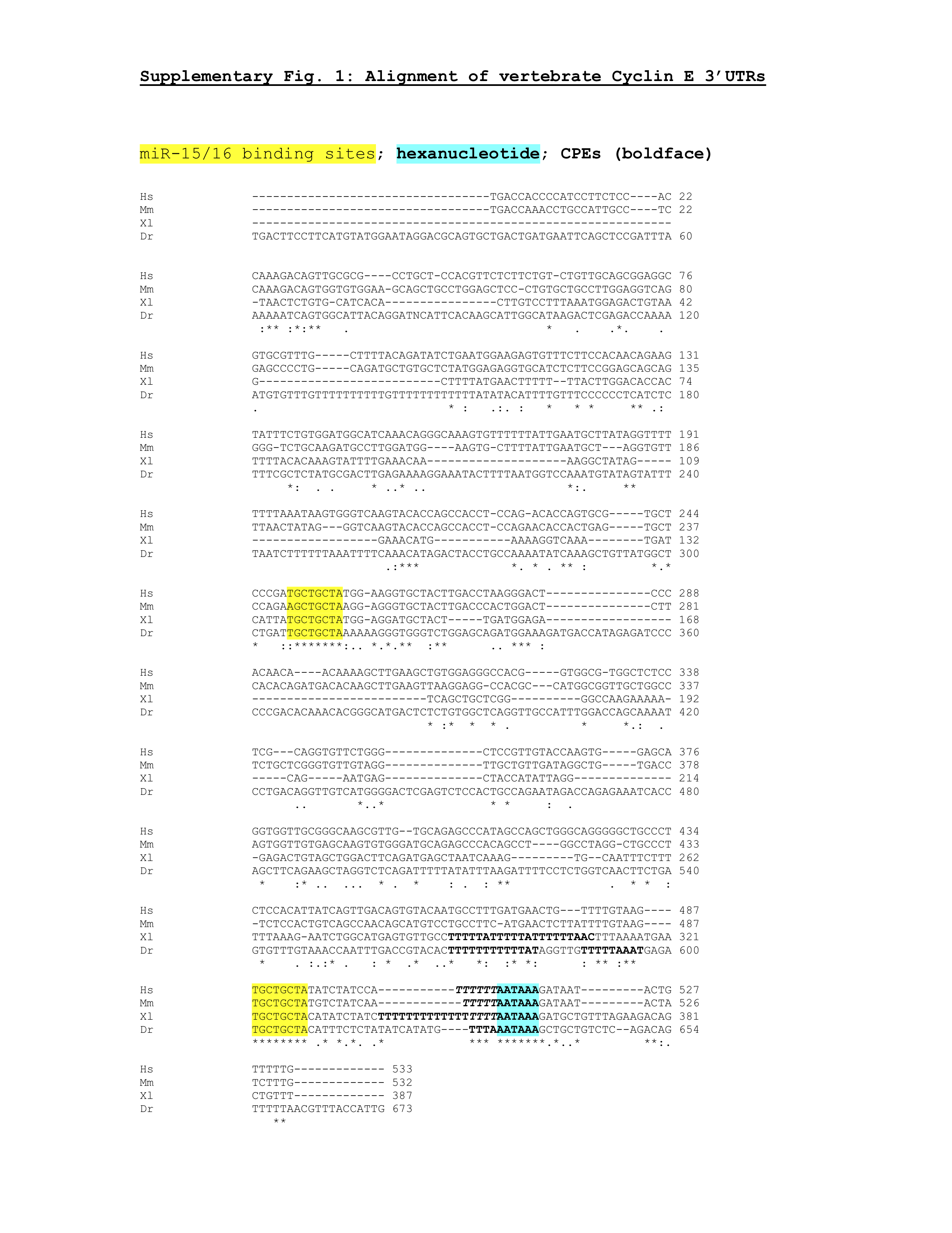

Supplement: S1 Fig — CPE sequences are in bold and italic (overlapping CPE), the nuclear hexanucleotide is bold and blue, and the two miR-15/16 binding sites are coloured yellow. Accession numbers as follows: Hs BC035498; Mm NM_007633; Xl3.1-IMAGE:6638064.5.5; Dr X83594. (TIF) [file pone.0146792.s001.tif]

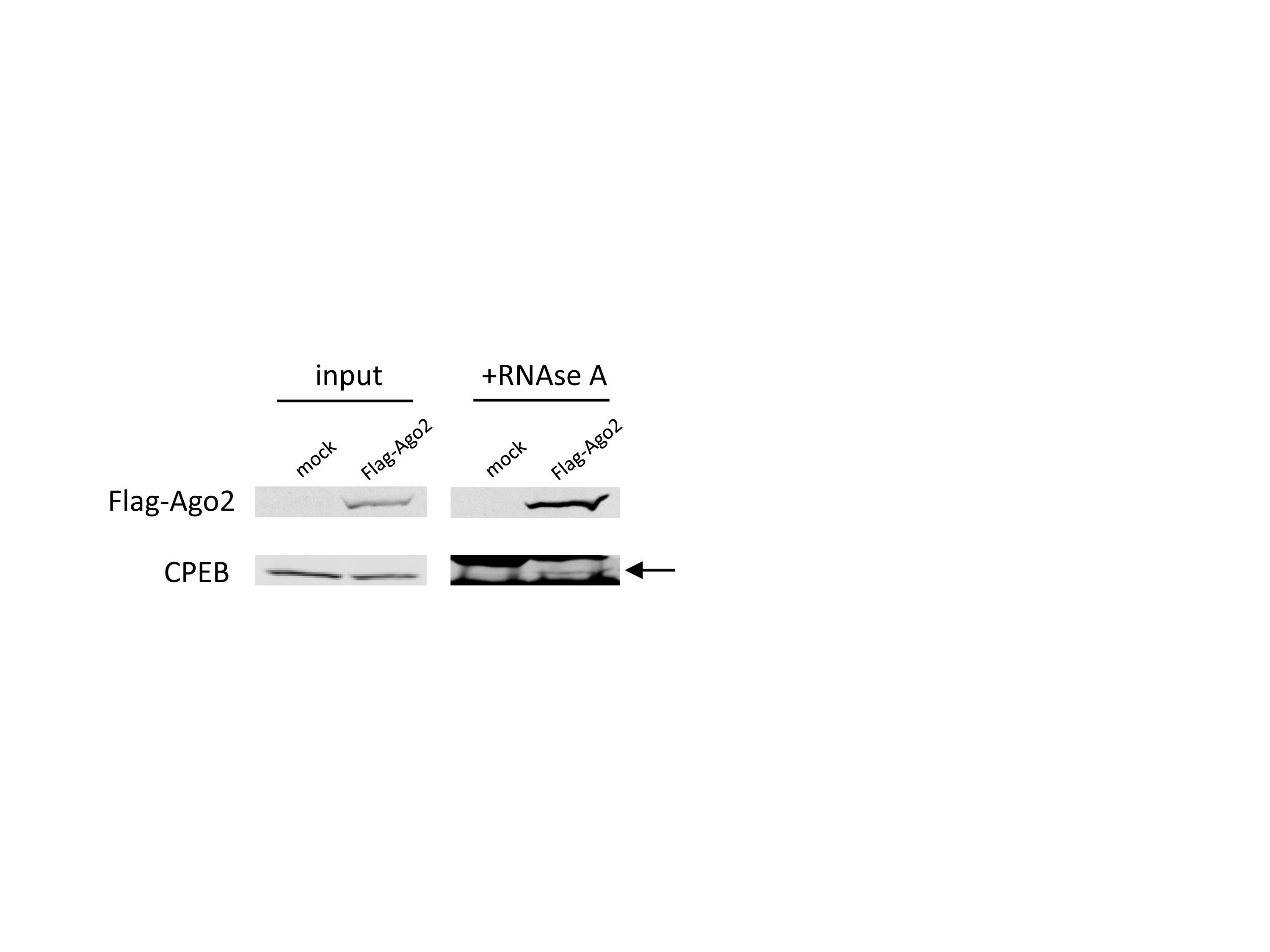

Supplement: S2 Fig — Oocytes were injected with FLAG-Ago2 mRNA, and the resulting lysates were subjected to immunoprecipitation with anti-FLAG magnetic beads in the presence of RNAse A. Input represents 10% of the immunoprecipitated fractions. Western blotting was performed with anti-FLAG or -CPEB1 antibodies. The arrow indicates the CPEB band. (TIF) [file pone.0146792.s002.tif]
