# Supplementary material for: CPEB and miR-15/16 Co-Regulate Translation of Cyclin E1 mRNA during Xenopus Oocyte Maturation
Source: PLoS One. 2016 Feb 1;11(2):e0146792. doi: 10.1371/journal.pone.0146792 (PMC4734764; doi:10.1371/journal.pone.0146792)
Supplement: S1 Table — (PDF) [file pone.0146792.s003.pdf]

Primers used in this study:

Name	Sequence (5'-3')
JA14	GATCCTAAATAGTGTATTGTGTTTTTAATGTTTTACTGGTTTTAATAAAGCTCATTTTAACATGG
JA15	AATTCCATGTTAAAATGAGCTTTATTAAAACCAGTAAAACATTAAAAACACAATACACTATTTAG
JA17	ATCGGGAGCTCCAGTGCTTTAACTCTGTGC
JA18	GCTAGGTACCAAAAAAAAACAGCTGCTTTCTAAACAGC
JA19	GCTACATATCTATCTTTGTTGTTGTTTTTAATAAAGATGCTGTTTAGAAGACAGCTGTTTTTTTTTG GTAC
JA20	CAAAAAAAAACAGCTGTCTTCTAAACAGCATCTTTATTAAAAACAACAACAAAGATAGATATGTA GCAG
JA21	GCATGAGTGTTGCCTTGGTATTTTTATTTTTTAAC
JA22	GTTAAAAAATAAAAATACCAAGGCAACACTCATGC
JA23	GCCTTGGTATTGGTATTTTTTAAC
JA24	GTTAAAAAATACCAATACCAAGGC
JA25	GGTATTGGTATTTGGTAACTTTAAAATG
JA26	CATTTTAAAGTTACCAAATACCAATACC
JA29	CTTTTTTTTTTTTTGGAATAAAGATGCTG
JA30	CAGCGTCTTTATTCCAAAAAAAAAAAAAG
JA31	CTTTATAATTTATTGACTTTGTTGTTTTTGGTATCTTATTGTC
JA32	GACAATAAGATACCAAAAACAACAAAGTCAATAAATTATAAAG
miR-16-1 mut for	GGTCAAATGATCATTAAGCAGGTATGGAGGATGCTACTTG
miR-16-1 mut rev	CAAGTAGCATCCTCCATACCTGCTTAATGATCATTTGACC
miR-16-2 mut for	CTTTAAAATGAAAGCAGGTACATATCTATC
miR-16-2 mut rev	GATAGATATGTACCTGCTTTCATTTTAAAG
Fluc Ccne1 Nhe mutF	GAAGACAGCTGTTTTTTTTTGCTAGCGGATCCCCTAAAAAAAAAAAAAAAAAAAAAAAAAG
Fluc Ccne1 Nhe mutR	CTTTTTTTTTTTTTTTTTTTTTTTTTAGGGGATCCGCTAGCAAAAAAAAACAGCTGTCTTC
Fluc EcoRI mutF	CAATTGCACTGATAATGAACTCCTCTGGATCTACTG
Fluc EcoRI mutR	CAGTAGATCCAGAGGAGTTCATTATCAGTGCAATTG
hRluc qPCR For	TCCGCAACTACAACGCCTAC
hRluc qPCR Rev	GGAACTTCTTAGCTCCCTCGAC
Fluc qPCR For	TCGCCAGTCAAGTAACAAC
Fluc qPCR Rev	ACTTCGTCCACAAACACAA
Cyclin E1 qPCR For	GCGTTTCTCCTCTTCCAC
Cyclin E1 qPCR Rev	ATGTTCCTCCAGACATCATCCT
GAPDH qPCR For	TGCATCCTGCACTACAAAC
GAPDH qPCR Rev	GTCCCTCAACAATGCCAAA
Universal Rev miRNA qPCR	GCGAGCACAGAATTAATACGACTC
XL-miR-15b miRNA qPCR	TAGCAGCACATCATGATTTGC
XL-miR-16a miRNA qPCR	TAGCAGCACGTAAATATTGGT
U1b snRNA qPCR	GACTGCGTTCGCGCTTTC
5S rRNA qPCR	GAATACCAGGTGTCGTAGGC
U2 snRNA qPCR	GTACCTCCAGGACCGGTG
piRNA-XL-MT3744	GTTCCTCACCGTTCCTGGT
